# Association between 45T/G Polymorphism of Adiponectin Gene and Coronary Artery Disease in an Iranian Population

**DOI:** 10.1100/tsw.2011.3

**Published:** 2011-01-05

**Authors:** Somaye Sabouri, Majid Ghayour Mobarhan, Mohsen Moohebati, Mitra Hassani, Jamal Kassaeian, Farnoosh Tatari, Fatemeh Mahmoodi-kordi, Habib A. Esmaeili, Shima Tavallaie, Roghayeh Paydar, Amirhossein Sahebkar, Shahireh Omidvar Tehrani, Gordon Ferns, Javad Behravan

**Affiliations:** ^1^ Biotechnology Research Center and School of Pharmacy, Mashhad University of Medical Science (MUMS), Mashhad, Iran; ^2^ Azarbaijan University of Tarbiat Moallem, Tabriz, Iran; ^3^ Cardiovascular Research Center, Avicenna Research Institute, MUMS, Mashhad, Iran; ^4^ Department of Nutrition and Biochemistry, Faculty of Medicine, MUMS, Mashhad, Iran; ^5^ Cardiovascular Genetics Research Unit, Henri Poincaré University, Nancy, France; ^6^ Department of Statistics, Faculty of Medicine, MUMS, Mashhad, Iran; ^7^ Post Graduate Medical School, University of Surrey, Guildford, Surrey, UK

## Abstract

A single nucleotide polymorphism (SNP) in the adiponectin gene, 45T/G, has been reported in relation to a number of metabolic disorders, including obesity, insulin resistance, and diabetes. However, previous studies on the association between this SNP and the presence of coronary artery disease (CAD) have been few, with no report from Iranian subjects. The present study set out to investigate the association between this SNP and CAD in an Iranian population. Among 464 patients (age: 18–75 years), recruited from individuals who underwent coronary angiography, 135 patients had less than 50% reduction of coronary artery diameter and were classified as the CAD- group and 329 patients had more than 50% reduction of coronary artery diameter and were classified as the CAD+ group. The last group was divided into single-vessel disease (n = 86), two-vessel disease (n = 111), and three-vessel disease (n = 132). Healthy subjects (n = 106) who did not have any history of heart diseases were also recruited as the control group. All subjects were genotyped for the 45T/G polymorphism using the polymerase chain reaction-restriction fragment length polymorphism (PCR-RFLP) technique. A significantly higher frequency of the TG genotype and G allele, which was paralleled by a lower frequency of the TT genotype and T allele, was observed in both CAD+ and CAD- patients when compared with the control group (*p* ≤ 0.001). There was no significant difference in the genotype distribution and allele frequencies between CAD+ and CAD- patients, and also between different subgroups of patients based on the number of stenosed vessels (*p* > 0.05). Our findings indicate that the presence of the G allele at the position +45 of the adiponectin gene may be associated with the risk of CAD in our study population. While we found no significant difference in the genotype distribution and allele frequencies between patients with angiography+ and angiography, this may be because the 50% stenosis cut-off does not discriminate sufficiently between individuals with and without significant coronary disease.

### INTRODUCTION

Adipose tissue is currently considered as an important endocrine organ that secretes multiple biologically active adipocytokines, such as leptin, acylation-stimulating protein (ASP), tumor necrosis factor-α (TNF-α), plasminogen activator inhibitor-1 (PAI-1), and interleukin-6[1,2]. Another important adipocytokine is adiponectin, which is a collagen-like, 247-amino-acid protein exhibiting antiatherogenic, anti-inflammatory, and antidiabetic properties[3,4,5,6]. This protein is highly and specifically expressed in adipose cells and is abundantly present in human plasma, with a concentration ranging from 3 to 30 μg/ml, thus accounting for 0.01% of total plasma protein[7,8,9]. A growing body of evidence indicates that adiponectin may exert favorable protective effects against cardiovascular diseases and is beneficial for the treatment or prevention of atherosclerosis, cardiac hypertrophy, fibrosis, and remodeling and myocardial injury[10,11,12,13,14,15]. The cardiovascular protective effects of adiponectin could be ascribed to different mechanisms, such as stimulation of angiogenesis, endothelial cell migration and differentiation, prevention of endothelial apoptosis, and reduction of the expression of TNF-α and vascular cell adhesion molecule-1[16,17,18,19]. In addition, plasma levels of adiponectin have been reported to be negatively associated with numerous cardiovascular risk factors, including triglycerides, LDL cholesterol, systolic and diastolic blood pressure, fasting plasma glucose, insulin, body mass index (BMI), atherogenic index, and apo B, but positively associated with serum HDL cholesterol or apo A-I levels[20,21].

The adiponectin gene (APM1, NM 004797) is located on chromosome 3q27, spans approximately 15.8 kb, and consists of three exons and two introns[22,23]. Various single nucleotide polymorphisms (SNPs) have been identified in the adiponectin gene. One of these SNPs, 45T/G, in exon 2 of the adiponectin gene, has been frequently reported in association with obesity, insulin resistance, serum adiponectin level, and type 2 diabetes mellitus, but some of the results have been controversial[24,25,26, 27,28,29]. Although the previously reported associations between the 45T/G polymorphism and obesity, insulin resistance, and diabetes may pose this SNP as a potential genetic marker or risk factor for coronary artery disease (CAD), there is still a lack of data on the association of the genetic variants of this SNP with the occurrence of CAD.

To our knowledge, genetic polymorphisms of the adiponectin gene in an Iranian population have not yet been studied. In addition, there is very little information about the genetic susceptibility to CAD in an Iranian population. Therefore, in the present study, we sought to determine the association between +45T/G SNP and the presence of CAD in a group of Iranian subjects.

### METHODS

#### Subjects

Four hundred and sixty-four patients were recruited from individuals aged 18–75 years who underwent coronary angiography at Ghaem Medical Center, Mashhad, Iran. Patients with acute infectious disease, heart disease, renal disease, pregnancy, or history of past angiography were excluded from the study population. The presence of CAD was defined as a >50% reduction of coronary artery diameter. Patients were classified according to the number of significantly stenotic vessels into angiographically normal (n = 135), one-vessel (n = 86), two-vessel (n = 111), and three-vessel (n = 132) diseased groups. Healthy control subjects (n = 106) were randomly selected from among individuals who visited affiliated hospitals or clinics, but did not have any history of heart diseases.

The study protocol was approved by the Mashhad University of Medical Sciences (MUMS) ethics committee and written informed consent was obtained from each participant.

### Anthropometric and Other Measurements

Anthropometric parameters, including weight, height, BMI, waist circumference, hip circumference, and waist/hip ratio, as well as systolic and diastolic blood pressures, were measured as previously described[30].

### Routine Biochemical Analysis

A full-fasted lipid profile was determined for each subject. Serum lipid and fasting blood sugar (FBS) concentrations were measured by enzymatic methods.

### Genotyping

Genomic DNA was extracted from frozen whole blood with the use of a Biogen kit (Iran). Fragments comprising the 45T/G of the adiponectin gene sequence were amplified by polymerase chain reaction (PCR) using the forward primer 5‘ – GAA GTA GAC TCT GCT GAG ATG G – 3‘ and the reverse primer 5‘ – TAT CAG TGT AGG AGG TCT GTG ATG - 3‘ under the conditions described by Xita et al.[31]. Briefly, for a 50-µl PCR, the reaction contained 200 ng genomic DNA, 200 µmol/l of each of the dNTPs (dATP, dCTP, dGTP, and dTTP), 250 ng of each primer, 1.5 mmol/l magnesium chloride, and 1 U *Taq* DNA polymerase (MBI Fermentas)[32]. PCR amplification consisted of an initial 5-min denaturation at 95ºC, followed by 35 cycles of denaturation at 94ºC for 60 sec, annealing at 58ºC for 45 sec, and extension at 72ºC for 45 sec[31]. The terminal extension was performed at 72ºC for 5 min. The PCR products were digested with *SmaI* (Fermentas Life Sciences, Lithuania) and the fragments were separated by electrophoresis on 2% ethidium bromide–stained agarose gel and visualized directly under ultraviolet illumination. Undigested 372-bp fragment indicated the presence of the T allele; heterozygous TG were at 372, 219, and 153 bp; and appearance of two bands at 219 and 153 bp represented the G allele in the 45T/G genotypes of subjects and healthy control groups.

### Statistical Analysis

All statistical analyses were performed using the SPSS for Windows™, version 11.5 software package (SPSS, Chicago, IL, USA). Data were expressed as means ± SD for normally distributed data, or median and interquartile range for non-normally distributed data. The statistical difference in genotype distribution and allele frequencies between the CAD+, CAD-, and control groups were assessed by the $\chi^2$ test. Other variables were compared using one-way ANOVA (for normally distributed variables) or Kruskal-Wallis test (for non-normally distributed variables). Compliance of genotypes with the Hardy-Weinberg equilibrium in each group was assessed by $\chi^2$ test with one degree of freedom. A two-sided $p$ value < 0.05 was considered statistically significant.

## RESULTS

### Demographic Characteristics

Of the total patients who underwent coronary angiography 54.2% were male and 45.8% were female. According to the results of coronary angiography, 84.2% of males and 55.9% of females were diagnosed with CAD. Therefore, the prevalence of CAD was found to be significantly higher in males compared with females ($p < 0.001$). There was a significant difference among the three groups in their age ($p < 0.001$). However, the groups did not differ significantly in their BMI and waist/hip ratio, except for a significant increase in the waist/hip ratio in the CAD+ group compared with the CAD- group ($p < 0.001$). The family history of coronary heart disease (CHD), stroke, diabetes mellitus, and peripheral vascular disease (PVD) was significantly higher in patients compared with controls ($p < 0.001$), but no significant difference was observed in the family history of the mentioned diseases between the CAD+ and CAD- groups ($p > 0.05$). The prevalence of hypertension, hyperlipidemia, angina pectoris, and heart failure did not differ between the CAD+ and CAD- groups ($p > 0.05$), but myocardial infarction and diabetes mellitus were more frequent among the CAD+ compared with CAD- patients ($p < 0.001$ and $p < 0.05$, respectively). In regard to the lipid profile, both the CAD+ and CAD- patients had higher serum triglycerides compared with the controls, as expected ($p < 0.01$). However, the CAD+ and CAD- groups had significantly higher serum levels of HDL-C and lower levels of total cholesterol and LDL-C in comparison with the control group ($p < 0.05$). No significant difference was observed in lipid profile parameters between CAD+ and CAD- patients ($p > 0.05$). Finally, both CAD+ and CAD- patients had significantly higher systolic blood pressure and fasting blood glucose than controls ($p < 0.001$). The mean fasting blood glucose was also significantly higher among CAD+ compared with CAD- patients ($p < 0.01$). General characteristics of study subjects are summarized in Table 1.

### Association between SNP 45T/G and Incidence of CAD

*In vitro* DNA amplification of the adiponectin gene using the specific primers resulted in a 372-bp DNA product. On digestion of the amplified fragments (amplicons) with *SmaI* restriction endonuclease, DNA fragments of 372 (TT), 372, 219, 153 (TG), and 153 (GG) bp length were observed. Thus, each sample revealed one of three different electrophoretic patterns.

Over all, out of a total of 464 patients who were recruited for the present study, the frequencies of the TT, TG, and GG genotypes were 77.2% (358 cases), 22.2% (103 cases), and 0.6% (three cases), respectively. Among the 106 control subjects, the frequencies of the TT and TG genotypes were 94.3% (100 cases) and 5.7% (six cases), respectively, while there was no case of the GG genotype. The allele frequencies were 0.883 and 0.117 in the patients and 0.972 and 0.028 in the control group for the T and G alleles, respectively. Genotype frequencies were in Hardy-Weinburg equilibrium ($p > 0.05$). A significantly higher frequency of the TG genotype, which was paralleled by a lower frequency of the TT genotype, was observed in both CAD+ and CAD- patients when compared with the control group ($p \leq 0.001$, Table 2). In regard to the allele frequencies, the frequency of the G allele was significantly higher in the CAD+ and CAD- groups compared with the control group ($p < 0.001$, Table 2). In contrast, the T allele was more frequent in the control group ($p < 0.001$, Table 2). However, there was no significant difference in the genotype distribution and allele frequencies between CAD+ and CAD- patients ($p > 0.05$, Table 2). In regard to the number of stenosed vessels in the CAD+ patients, no significant difference was detected among patients with 1, 2, and 3 stenosed vessels, neither in the genotype nor in the allele frequencies ($p > 0.05$, Table 3).

**TABLE 1**
**General Characteristics of CAD+, CAD-, and Control Groups**

| | | CAD+ | CAD- | Control |
|---|---|---|---|---|
| N | | 329 | 135 | 106 |
| Age (years) | | 58.4 ± 10.6***♣♣♣ | 52.4 ± 11.7*** | 47.6 ± 9.4 |
| Male (%) | | 64.1♣♣♣ | 29.7*** | 56.3 |
| Height (cm) | | 160.4 ± 11.1 | 158.2 ± 9.9 | 160.7 ± 10.8 |
| Weight (kg) | | 70.1 ± 14.3♣ | 66.9 ± 13.2* | 69.5 ± 14.0 |
| BMI (kg/m$^2$) | | 27.6 ± 8.6 | 26.8 ± 5.5 | 27.5 ± 5.6 |
| Waist circumference (cm) | | 90.2 ± 12.0**♣ | 87.2 ± 12.7*** | 94.6 ± 11.6 |
| Hip circumference (cm) | | 94.5 ± 11.8*** | 94.84 ± 12.01*** | 102.6 ± 9.1 |
| Waist/hip ratio | | 0.9 ± 0.1♣♣♣ | 0.9 ± 0.1 | 0.9 ± 0.1 |
| TC (mg/dl) | | 172.8 ± 50.1* | 170.0 ± 49.5** | 186.6 ± 33.3 |
| TG (mg/dl) | | 129.0 (94.2–186.0)*** | 114.0 (83.0–171.0)** | 101.0 (71.0–141.0) |
| HDL-C (mg/dl) | | 43.1 ± 13.0* | 44.9 ± 12.0** | 39.8 ± 8.4 |
| LDL-C (mg/dl) | | 100.0 ± 37.1*** | 100.3 ± 37.9*** | 120.3 ± 36.1 |
| FBS (mg/dl) | | 123.0 ± 60.0***♣♣ | 107.2 ± 42.5*** | 79.5 ± 12.0 |
| SBP (mmHg) | | 144.5 ± 30.3*** | 142.8 ± 25.1*** | 119.2 ± 17.9 |
| DBP (mmHg) | | 78.6 ± 14.0 | 75.6 ± 14.4 | 78.6 ± 11.5 |
| Hypertension (%) | | 48.3*** | 46.1*** | 0 |
| Hyperlipidemia (%) | | 40.1*** | 33.5*** | 0 |
| Angina pectoris (%) | | 3.7*** | 3.0*** | 0 |
| Myocardial infarction (%) | | 23.8***♣♣♣ | 7.9*** | 0 |
| Diabetes mellitus (%) | | 27.5***♣ | 19.4*** | 0 |
| Heart failure (%) | | 4.0*** | 7.3*** | 0 |
| Family history of | CHD (%) | 23.5*** | 23.6*** | 0 |
| | Stroke (%) | 8.4*** | 9.7*** | 0 |
| | DM (%) | 12.4*** | 13.9*** | 0 |
| | PVD (%) | 3.5*** | 4.2*** | 0 |

Values are expressed as mean ± SD, or median and interquartile range. Comparisons were made by the $\chi^2$ test (for categorical data), one-way ANOVA, and Kruskal-Wallis test (for numerical data). *$p < 0.05$, **$p < 0.01$, ***$p < 0.001$: compared with the control group; ♣$p < 0.05$, ♣♣$p < 0.01$, ♣♣♣$p < 0.001$: compared with the CAD- group. BMI: body mass index; TC: total cholesterol; TG: triglycerides; HDL-C: high-density lipoprotein cholesterol; LDL-C: low-density lipoprotein cholesterol; FBS: fasting blood sugar; SBP: systolic blood pressure; DBP: diastolic blood pressure; CHD: coronary heart disease; DM: diabetes mellitus; PVD: peripheral vascular disease.

### DISCUSSION

Identification of polymorphisms associated with genetic susceptibility to CAD offers the potential for clinical preventive interventions. To our best knowledge, investigations concerning the association between adiponectin polymorphisms and cardiovascular outcomes have not been performed as much as those performed in relation to diabetes or insulin resistance. Nevertheless, the findings about SNP 45T/G have been inconsistent.

Initially, in a cross-sectional study on 477 Caucasians with type 2 diabetes from France and Switzerland (162 cases with CAD and 315 controls), Lacquemant and coworkers reported a significant association between SNP 45T/G and CAD[33]. However, this positive finding has not been confirmed by subsequent

**TABLE 2**
**Genotype Distribution and Allele Frequencies of SNP +45T/G in CAD+, CAD-, and Control Subjects**

| | CAD+ | CAD- | Control |
|---|---|---|---|
| Genotype distribution | | | |
| TT | 253 (76.9)*** | 105 (77.8)*** | 100 (94.3) |
| TG | 74 (22.5)*** | 29 (21.5)** | 6 (5.7) |
| GG | 2 (0.6) | 1 (0.7) | 0 (0) |
| Allele frequencies | | | |
| T | 0.881*** | 0.885*** | 0.972 |
| G | 0.119*** | 0.115*** | 0.028 |

Values are expressed as number (%) for genotypes and frequency for alleles. Comparisons were made by the $\chi^2$ test. ***p* < 0.01, ****p* < 0.001: compared with the control group.

**TABLE 3**
**Genotype Distribution and Allele Frequencies of SNP +45T/G in the Subgroups of CAD+ Patients, CAD- Patients, and Control Subjects**

| | SVD | 2VD | 3VD | CAD- | Control |
|---|---|---|---|---|---|
| Genotypes | | | | | |
| TT | 64 (74.4)*** | 84 (75.7)*** | 105 (79.5)** | 105 (77.8)*** | 100 |
| TG | 22 (25.6)*** | 26 (23.4)*** | 26 (19.7)** | 29 (21.5)** | 6 |
| GG | 0 | 1 (0.9) | 1 (0.8) | 1 (0.7) | 0 |
| Alleles | | | | | |
| T | 0.872*** | 0.874*** | 0.894** | 0.885*** | 0.972 |
| G | 0.128*** | 0.126*** | 0.106** | 0.115*** | 0.028 |

Values are expressed as number (%) for genotypes and frequency for alleles. Comparisons were made by the $\chi^2$ test. ***p* < 0.01, ****p* < 0.001: compared with the control group. SVD: single-vessel disease; 2VD: two-vessel disease; 3VD: three-vessel disease.

studies. In a survey of 879 male diabetic Americans from the Health Professional Follow-up Study, Qi et al. failed to find any significant associations between +45 variants and cardiovascular risk[26]. Similarly, the same authors did not find any significant association between this SNP and the risk of cardiovascular disease in 989 female diabetic Americans from the Nurses' Health Study[34]. Moreover, Bacci and colleagues did not find any significant difference in genotype distribution at position +45 between CAD and control subjects in a total of 376 Italian subjects with type 2 diabetes[35].

As stated, all of the above studies have been performed in diabetic subjects. In regard to the nondiabetic populations, Filippi et al. did not find any significant difference in the genotype and allele frequencies of SNP 45T/G between patients with CAD and control subjects in their study of 595 Caucasians from Italy[36]. In another recent study, SNP 45T/G was not found to be related to the risk of CHD, either in males (798 subjects from the Health Professional Follow-up Study) or in females (747

subjects from the Nurses' Health Study). As well, in the same study, no significant association was observed between the mentioned SNP and risk of coronary artery bypass graft surgery or percutaneous transluminal coronary angioplasty in 564 male individuals[37]. Likewise, Jung and colleagues did not find any significant association between this SNP and the presence of CAD in their survey of 156 Korean subjects[38]. This finding was in accordance with a previous report on 902 nondiabetic Korean subjects for whom no significant genotype effects of SNP 45T/G on cardiovascular risk factors, including blood pressure, anthropometric parameters, lipid profile, serum glucose, homeostasis model assessment of insulin resistance (HOMA-IR), circulating adiponectin, plasma C-reactive protein (CRP), and urinary prostaglandin F2α concentrations, was observed[39].

For the evaluation of consistency between the results of the present work with those of aforementioned studies, the design of our study must be taken into consideration. Unlike most of the previous studies, we compared the SNP 45T/G variants among three groups: a CAD+ group with angiographically defined CAD; a CAD- group that comprised patients with chest pain and relatively high risk factors for CAD, but with <50% reduction in coronary artery diameter; and a control group that included healthy subjects without any personal or familial history of heart diseases or diabetes. The lack of significant difference in genotype distribution and allele frequency between CAD+ and CAD- patients in our study is to some extent in line with those studies performed in diabetic subjects, and failed to report any significant association between this SNP and cardiovascular diseases[27,34,35]. Since diabetic patients have higher risk and are more prone to develop cardiovascular disease, those diabetics who were not identified with cardiovascular diseases in previous studies could be considered almost the same as CAD- subjects in the present study who were also at high risk for cardiovascular disease. Our results are also consistent with the finding of Jung et al. who found no significant difference in SNP 45T/G variants between CAD+ and CAD- subjects[38], although the mentioned study was performed in an Asian population, not Caucasian. However, the significant differences in genotype distributions and allele frequencies between CAD+ patients and control subjects that was observed in the current study stand in contrast with previous reports by Lacquemant et al.[33], Filippi et al.[36], and Pischon et al.[37]. Also, the favorable lipid profile status of patients compared with controls is probably due to the consumption of statin drugs by patients. In addition, our results indicate that SNP +45 variants are not associated with the number of stenosed vessels and, therefore, degree of ischemia.

As a limitation of our study, we did not measure the serum levels of adiponectin, which could give us a better understanding about the mechanism. However, the impact of 45T/G SNP on the circulating levels of adiponectin has been the matter of several previous studies, but the results have been inconsistent[28,39]. Furthermore, a recent systematic meta-analysis failed to report any significant association between this SNP and adiponectin levels[40]. Therefore, mechanisms other than alteration of serum adiponectin levels should be taken into consideration for the observed effect of this SNP. Finally, confirmation of our findings in additional larger populations would be helpful to identify those subjects who are at cardiovascular risk, which implies early and appropriate intervention.

### CONCLUSION

The present study investigated the association between a polymorphism in the adiponectin gene and CAD in an Iranian population. Our results demonstrated that the presence of the TG genotype or G allele at the position +45 of the adiponectin gene may be associated with the risk of CAD, but it remains to be clarified whether this impact is independent or due to a linkage disequilibrium with another mutation. Although we found no significant difference in the genotype distribution and allele frequencies between patients with angiography+ and angiography-, or between different subgroups of patients based on the number of stenosed vessels, this may be because of the cut-off value of 50% stenosis used to define the presence of coronary disease. It is possible that some of the subjects with <50% coronary stenosis will go on to develop overt coronary disease over time.

### ACKNOWLEDGMENTS

The authors acknowledge the Research Council of Mashhad University of Medical Sciences for the financial support of the present work. We also sincerely thank all of the subjects who participated in the study.
